# Antiseptic-Loaded Casein Hydrogels for Wound Dressings

**DOI:** 10.3390/pharmaceutics15020334

**Published:** 2023-01-19

**Authors:** Leonor Vasconcelos Garcia, Diana Silva, Maria Madalena Costa, Henrique Armés, Madalena Salema-Oom, Benilde Saramago, Ana Paula Serro

**Affiliations:** 1 Centro de Química Estrutural, Institute of Molecular Sciences, Departamento de Engenharia Química, Instituto Superior Técnico, Universidade de Lisboa, Av. Rovisco Pais, 1049-001 Lisboa, Portugal; 2 Hospital Veterinário de S. Bento, Rua de S. Bento, 358-A, 1200-822 Lisboa, Portugal; 3 Faculdade de Medicina Veterinária, Universidade Lusófona, Campo Grande, 376, 1749-024 Lisboa, Portugal; 4 Centro de Investigação Interdisciplinar Egas Moniz (CiiEM), Instituto Universitário Egas Moniz, 2829-511 Caparica, Portugal

**Keywords:** wound dressings, casein, hydrogel, antiseptics, drug release

## Abstract

Chronic wound treatment accounts for a substantial percentage of the medical expenses worldwide. Improving and developing novel wound care systems can potentially help to handle this problem. Wound dressings loaded with antiseptics may be an important tool for wound care, as they inhibit bacterial growth at the wound site. The goal of the present work was to investigate the potential of using casein hydrogel dressings loaded with two antiseptic drugs, Octiset^®^ or polyhexanide, to treat chronic wounds. Casein-based hydrogels are inexpensive and have several properties that make them suitable for biomedical applications. Two types of casein were used: casein sodium salt and acid casein, with the formulations being labelled CS and C, respectively. The hydrogels were characterised with respect to their physical properties (swelling capacity, water content, morphology, mechanical resistance, and stability), before and after sterilisation, and they showed adequate values for the intended application. The hydrogels of both formulations were able to sustain controlled drug-release for, at least, 48 h. They were demonstrated to be non-irritant, highly haemocompatible, and non-cytotoxic, and revealed good antimicrobial properties against *Staphylococcus aureus* and *Pseudomonas aeruginosa*. Steam-heat sterilisation did not compromise the material’s properties. The in vivo performance of C hydrogel loaded with Octiset^®^ was evaluated in a case study with a dog. The efficient recovery of the wounds confirms its potential as an alternative for wound treatment. To our knowledge, this is the first time that wound dressings loaded with Octiset^®^, one of the most efficient drugs for wound treatment, were prepared and tested.

## 1. Introduction

Chronic wounds are a huge challenge for wound care professionals because they may take a long time to heal and frequently involve clinical complications [1,2]. Ulcers are the most common type of chronic wounds, and include diabetic foot ulcers, pressure ulcers, and venous leg ulcers. Among patients with diabetes, 2% to 3% will develop a foot ulcer each year, and approximately 15% will develop a foot ulcer during their lifetime [3,4]. Pressure ulcers have long been recognised as a disease entity [5]. The majority of leg ulcers can be related to venous disease, but other causes are possible, such as immobility, obesity, trauma, arterial disease, vasculitis, diabetes, and neoplasia [6,7]. Surgeries on the legs, such as a hip replacement or knee replacement, can also be associated with the occurrence of leg ulcers [8]. The normal wound healing process involves a set of sequential events: rapid haemostasis, appropriate inflammation, proliferation, and maturation [9]. Chronic wounds become trapped in the inflammatory and proliferative phases, which delays healing. The epidermis fails to migrate across the wound tissue, and there is hyperproliferation at the wound margins, which interferes with normal cellular migration over the wound bed [10]. In order to optimise wound healing, a clean, healthy granulating wound base must be kept, eventual infection must be treated, and the wound should be covered with an appropriate dressing able to ensure that the wound has an adequate level of moistness [11].

Modern wound dressings have been designed to facilitate wound healing rather than just covering it [12]. Ideally, a wound dressing should remain in contact with the wound, allowing gas exchange, adequate temperature, and an electrical gradient. Moreover, it should be non-toxic, non-allergic, and non-adherent, while preventing the proliferation of pathogens [13]. Currently available commercial dressings for advanced treatment of wounds are often inadequate, may cause damage to the skin due to adherence issues, and are generally expensive. Furthermore, the design of new and more efficient alternatives requires the conjugation of appropriate materials with optimised properties, together with therapeutic strategies to obtain dynamic wound healing [14]. Accordingly, hydrogels are excellent dressing materials for several types of wounds, due to their particular properties such as high similarity to the biological tissues, hydrophilic nature, water content, and adequate flexibility [15,16,17]. Hydrogels can be formulated using a wide range of polymers, including biopolymers of food origin. The benefits of hydrogels made from such biopolymers include safety, low cost, and wide availability. Among them, those based on casein, alone or in combination with other food-grade polymers, are of particular interest [18,19,20,21]. Casein-based hydrogels are biodegradable, biocompatible, renewable, easy to obtain, inexpensive, and nontoxic. These properties together with the ability to form networks of variable tensile strength and to encapsulate, protect, and release biomolecules led casein hydrogels to receive increasing attention from researchers in the area of biomaterials [22]. The incorporation of antiseptics into wound dressings potentiates the antimicrobial action of the dressing materials, preventing the occurrence of bacterial infections in the wound bed. Particular attention has been given to antiseptics, since they may have advantages over antibiotics: they have a broader spectrum of activity and unlike antibiotics, have multiple cellular targets. According to McDonnell and Russel [23], an increase in bacterial resistance to biocides (antiseptics and disinfectants) does not necessarily means its therapeutic failure.

Although casein-based hydrogels have already been investigated as platforms to release bioactive compounds [22], only a limited number of studies have evaluated drug-loaded dressings made of this type of hydrogels [24,25]; hence, further investigation is needed. The drugs tested in the referred works were gentamicin sulphate, an antibiotic [24], and allicin, a model antibacterial [25]. To our knowledge, there are no reports in the literature about the long-term use of wound dressings loaded with the most commonly used antiseptics in topical wound treatment [26,27,28,29,30].

**Figure 1:**
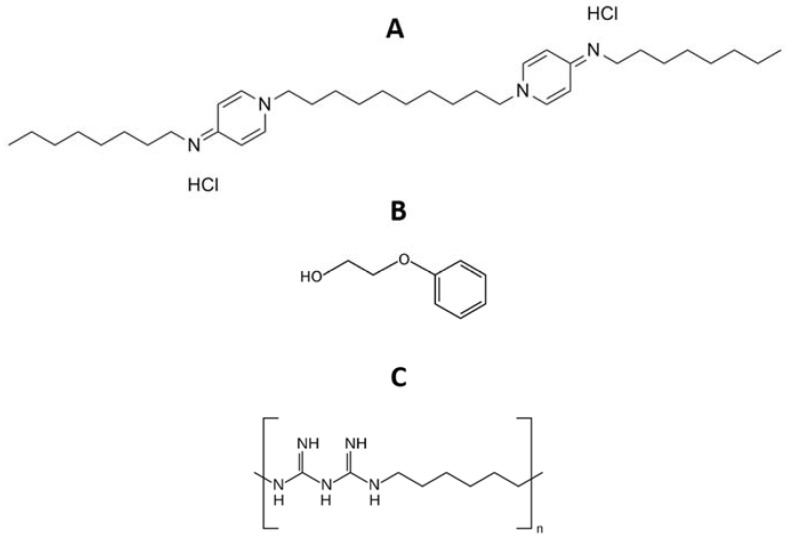
Chemical structure of (**A**) octenidine dihydrochloride, (**B**) 2-phenoxyethanol, and (**C**) polyhexanide.

The present work aimed to develop antiseptic-eluting casein-based hydrogels for wound dressings. The studied antiseptics were Octiset^®^ and polyhexanide. Octiset^®^ is a commercial solution, whose active ingredients are octenidine dihydrochloride (1 mg/mL) and 2-phenoxyethanol (20 mg/mL). Octenidine is a cationic surfactant effective against Gram-positive and Gram-negative bacteria. It features two non-interacting cationic active centres separated by a lengthy aliphatic hydrocarbon chain (Figure 1A), which facilitate its linkage with negatively charged surfaces of the microorganisms [31]. 2-Phenoxyethanol (Figure 1B) is an aromatic ether with a 2-hydroxyethyl group substituting on oxygen. Its known antimicrobial activity derives from the inactivation of malate dehydrogenase and uncoupling of oxidative phosphorylation. Interestingly, 2-phenoxyethanol has low inhibition effects on the resident skin bacteria [32]. Octiset^®^ is a new generation antiseptic with high efficacy for the treatment of skin wounds and mucous membranes. Polyhexanide (Figure 1C) is an antimicrobial polymeric biguanide, widely used for treating chronic wounds and burns. Its activity is often related to the attraction to negative charge phospholipids on the cells’ membrane, thereby impairing its function [33].

The composition of the produced hydrogels was optimised in order to increase their drug loading capacity. The swelling, the degradation profile, the mechanical behaviour, and the drug release of the different drug-loaded samples were assessed. Being materials of natural origin, the casein hydrogels are more susceptible to the sterilisation process, which may alter their characteristics in different ways [34]. Therefore, the effects of steam-heat sterilisation on the material properties were evaluated. Antimicrobial tests and other biological tests, such as cytotoxicity, haemocompatibility and irritability assays, were also carried out. Finally, a case study with a dog was conducted.

## 2. Materials and Methods

### 2.1. Materials

Casein sodium salt and acid casein, both from bovine milk, acrylamide (AAm), *N*,*N*’- methylenebisacrylamide (MBAAm), ammonium persulfate (APS), *N*,*N*,*N*’,*N*’ tetramethylenediamine (TEMED), *N*-(3-aminopropyl)methacrylamide hydrochloride (APMA), lysozyme from chicken egg white (40,000 units/ mg protein), phosphate buffered saline (PBS), NaCl, KCl, NaH_2_PO_4_, Dulbecco’s modified eagle’s medium (DMEM) (D5796), bovine calf serum, penicillin-streptomycin solution, sodium pyruvate, NIH/3T3 fibroblasts (ATCC^®^ CRL-1658/Sigma 93061524), trypsin EDTA (ethylenediaminetetraacetic acid) solution, dimethyl sulfoxide (DMSO), ethylenediaminetetraacetic acid solution (MTT solvent), and methanol were all purchased from Sigma (St. Louis, MO, USA). NaHCO_3_ was purchased from Panreac (Barcelona, Spain), and NaOH pellets (99%), from Merck (Darmstadt, Germany). Octiset^®^ was purchased from Schülke (Norderstedt, Germany), and polyhexanide (polyhexamethylene biguanide hydrochloride, PHMB) 94%, from Carbosynth (Berkshire, UK). Mueller–Hinton Agar and Mueller–Hinton Broth were purchased from Oxoid Ltd. (Hampshire, UK). Distilled and deionised (DD, 18 MΩcm, pH 7.7) water was obtained with a Millipore system (Millipore Merck, Darmstadt, Germany).

### 2.2. Hydrogels Preparation

Casein hydrogels were prepared via free radical polymerisation of AAm and coagulation of casein micelles. Two different formulations were used: formulation CS with casein sodium salt, and formulation C with acid casein.

The preparation of the casein hydrogels was based on the method proposed by Ma et al. [35]. First, PAAm chains were formed by radical polymerisation of AAm. Then, casein micelles that were formed by dissolution of casein in water lost their negative surface charge through acidification and coagulated, and were integrated in the PAAm chains. Further details of the synthetic process may be found in the above-mentioned reference.

For the formulation CS, the casein solution was prepared by dissolving 1 g of the casein sodium salt in 10 mL of DD water with magnetic stirring for 4 h. The pH of the solution was adjusted to 6 by the addition of NaOH solution (1 M). After dissolution, 2 g of AAm, 134 mg of APMA, and 1 mg of MBAAm were added to 5 mL of the casein solution. After stirring, 1 mg of APS and 0.5 μL of TEMED were added as a radical initiator and crosslinking accelerator for AAm, respectively. For the formulation C, the protocol was the same with an exception in the initial step, where the casein had to be dissolved in DD water with ≈20 μL of NaOH (10 M), given the very low solubility of this casein in pure water. The mixture was then magnetically stirred overnight. It is important to point out that the pH of the solution decreased as the casein started to dissolve, reaching a pH around 6 when it was fully dissolved.

The solutions were poured into glass moulds silanised as described previously [36]. Polymerisation was done by exposing the solutions to UV light (proMa, model UV Belichtungsgerät 2, Sande, Germany) for 4 h. Then, formulations CS and C were kept in the oven at 36 °C for 22 h and 6 h, respectively. The hydrogels were carefully removed from the moulds and washed in DD water for 3 days to eliminate the free radicals.

Hydrogel disks with 10 mm diameter were cut and dried in the oven for 6 h at 36 °C to be used in all tests, except the drug loading/release experiments, mechanical tests, and the cytotoxicity assay. Sterilisation of the hydrogels was carried out in an autoclave (Uniclave 88 from AJC, Cacém, Portugal) at 121 °C for 20 min.

### 2.3. Hydrogels Characterisation

#### 2.3.1. FTIR Analysis

The chemical structure of the dry non-loaded casein-based hydrogels was studied using Fourier transform infrared spectroscopy (FTIR), with attenuated total reflectance (ATR). We used a FTIR equipment (model Spectrum Two from PerkinElmer, Waltham, MA, USA) with a lithium tantalate (LiTaO_3_) mid-infrared (MIR) detector (signal/noise ratio 9300:1). The applied force was manually controlled, to ensure a good contact between the crystal (diamond crystal ATR accessory, model UATR Two) and the hydrogels. All spectra were collected at 4 cm^−1^ resolution and 8 scans of data accumulation and normalised using the OriginPro 8.5 software. Triplicates of each hydrogel (10 mm of diameter) were analysed.

#### 2.3.2. Swelling Ratio and Equilibrium Water Content

The swelling ratio (SR) of the hydrogels in four testing liquids, DD water, PBS, Octiset^®^, and polyhexanide solution in PBS (0.5 mg/mL), was determined using Equation (1). The equilibrium water content (EWC) was calculated only for the samples hydrated in DD water, through Equation (2) [37].

(1) $$SR\left(\%\right)=\frac{w_{h}-w_{d}}{w_{d}}\times 100$$

(2) $$EWC\left(\%\right)=\frac{w_{h}-w_{d}}{w_{h}}\times 100$$

The weight of the dried disks, *w_d_*, was determined, and they were then transferred into falcons (Labox, Enzymatic, Santo Antão do Tojal, Portugal) with 5 mL of the testing liquids. During the hydration process, the samples were carefully taken out of the solutions for several times, blotted with an absorbent paper, and weighted (*w_h_*). This was repeated until a constant weight was achieved. The assays were performed in triplicate.

#### 2.3.3. Degradation Assay

The hydrolytic degradation of the hydrogels was carried out in PBS, while the degradation was performed in simulated exudate solution in pseudo extracellular fluid (PECF) (0.68 g of NaCl, 0.229 g of KCl, 2.5 g of NaHCO_3_, and 0.4 g of NaH_2_PO_4_) containing lysozyme (1 mg/mL). The dried disks were weighted, and each disk was then immersed in 5 mL of the degradation solution at 34 °C, under agitation at 180 rpm. After 24 h and 48 h, the disks were washed by immersion in DD water and dried. The dried disks were weighed, and the weight loss was calculated through Equation (3), where *w*_0_ is the weight of the dried disks and *w*_24/48_ are the weights of the sample, after 24 h or 48 h in the degradation solutions, respectively, after drying [38]. The assay was done in quintuplicate.

(3) weight loss %=w0−w2448w0×100

#### 2.3.4. SEM

The hydrogel’s surface was observed using scanning electron microscopy (SEM). Prior to the SEM analysis, samples were lyophilised and coated with a gold/palladium film in a Polaron Quorum Technologies sputter coater and evaporator (Au/Pd). After coating, the disks were analysed with an Analytical SEM Hitachi S2400 (Hitachi Ltd., Tokyo, Japan). SEM images were obtained under 100×, 3000×, and 5000× magnifications. Cross-section images were obtained under 40× and 500× magnifications using samples cracked in liquid nitrogen. The assay was done in duplicate.

#### 2.3.5. Mechanical Tests

Tensile tests were performed using a texturometer (TA.XT Express Texture Analyser, Stable Micro Systems, Godalming, Surrey, UK). The hydrated hydrogels were cut using a special dumbbell-shape cutter (2.5 mm maximum width and 6 mm gauge length). The data were processed using the software TE32LiteExpress v. 6.1.15.0 (Godalming, Surrey, UK). A constant speed of 0.5 mm/s was applied. The obtained stress–strain curves allowed the calculation of the Young’s modulus and toughness. The range of strain considered for this assay was 0–20%, where stress and strain are proportionally dependent, and the curves presented a linear form [39]. The experiments were carried out in quadruplicate.

### 2.4. Drug Loading and Release

The hydrogels were loaded with the drugs by soaking in Octiset^®^ (1 mg/mL of octenidine dihydrochloride and 20 mg/mL of 2 phenoxyethanol, pH 6.4) or in polyhexanide solution in PBS (0.5 mg/mL, pH 7.5). Dried disks (with a diameter of 20 mm) were individually immersed in falcons containing 5 mL of the desired drug solution for 48 h at room temperature.

Drug release tests were performed in Franz diffusion cells [38]. The loaded disks were carefully blotted and placed surrounded by a rubber ring between the upper and lower parts of the cells, which were fixed with a clamp. The top side of the disk was left exposed to air in a confined environment. After mounting the six cells, the receptor chambers were filled with 6.5 mL of PBS, with special care to avoid air bubbles. The useful area of the samples in contact with the liquid was 76 mm^2^. To best mimic the in vivo conditions, experiments were done at normal human skin temperature (34 °C) [38]. At pre-determined times (every 30 min in the first hour, every hour for the remaining 7 h, and thereafter at 24 h and 48 h), 200 μL aliquots of the release solution were collected from each cell and the same volume of fresh PBS was refilled into the cell through the lateral tip. The absorbance of the collected solutions was analysed using UV-Vis spectroscopy (MultiskanTM GO Microplate Spectrophotometer, Thermo Scientific, Kandel, Germany) at characteristic wavelengths for each drug: 220 nm for 2-phenoxyethanol and 270 nm for octenidine dihydrochloride (i.e., the active components of Octiset^®^), and 220 nm for polyhexanide. From the absorbance values, the concentrations and the normalised cumulative mass released values were calculated.

In order to determine the amount of drug loaded into the hydrogel, a methanol extraction assay was performed following the protocol described in a previous work [40]. Briefly, drug-loaded samples were immersed in 3 mL of methanol inside glass vials. At pre-determined times (2 h, 4 h, 8 h, and 24 h), the disks were removed and placed in new vials with fresh methanol. The absorbance of the solution was assessed at each time point, the drug concentration was calculated, and the extracted drug mass was determined. The process was repeated until the methanol solution was free from the drugs. The experiments were carried out at least in triplicate.

### 2.5. Antibacterial Properties

The antibacterial properties of the hydrogels were evaluated against two bacteria: *Staphylococcus aureus* ATCC 25923 (Gram-positive) and *Pseudomonas aeruginosa* ATCC 15442 (Gram-negative), via turbidimetry. The experiment was performed under aseptic conditions (flow chamber from Bio Air Instruments, model AURA 2000 MAC 4 NF, Pero, Italy). Bacterial strains were grown for 24 h at 37 °C. An optical density of 1 McFarland (3 × 10^8^ bacteria/mL) was achieved for *Staphylococcus aureus* and of 0.5 McFarland (1.5 × 10^8^ bacteria/mL) for *Pseudomonas aeruginosa* by suspending the grown strains in 0.9% NaCl sterile solution. Mueller–Hinton broth medium was prepared and sterilised in the autoclave at 121 °C for 20 min. Each disk was carefully blotted with absorbent paper and individually placed in a 24-well plate. A total of 500 μL of the broth medium and 10 μL of the bacterial suspension were added to each well. For the positive control, 500 μL of the broth medium and 10 μL of the bacterial suspension were added to the well (without any sample), and for the negative control, only 500 μL of the broth medium was added. The plates were incubated at 37 °C for 24 h at 100 rpm. To analyse the results, 200 μL of each well solution was extracted, and the absorbance was measured using a spectrophotometer (Platos R 496 Microplate Reader, Labordiagnostik, Graz, Austria) at 630 nm. The assays were done in quadruplicate for non-loaded (hydrated in PBS) and drug-loaded (with Octiset^®^ or polyhexanide) CS and C hydrogels.

### 2.6. Biocompatibility Tests

#### 2.6.1. Irritation Assay (HET-CAM)

The Hen’s Egg test on the chorioallantoic membrane (HET-CAM) assay was performed to evaluate the potential irritation effect of both hydrogel formulations. The assay was done for drug-loaded and non-loaded samples. Fertilised hen’s eggs (Sociedade Agrícola da Quinta da Freiria, SA, Portugal) were incubated (Incubator, 56S, Nanchang Edward Technology Co., Ltd., Nanchang, China) at 37 °C ± 0.5 °C with 60 ± 5% of relative humidity. After incubating for 9 days, the shell was cut at the air pocket in the larger end of the egg, with a rotary saw (Dremel 3000 from Breda, Netherland), removed, and the inner membrane was hydrated with 0.9% NaCl. After hydration, this membrane was carefully removed in order to expose the chorioallantoic membrane (CAM).

Sterilised disks of the hydrogels were placed directly on the CAM for 5 min. Irritation of the membrane was evaluated by checking the appearance of lysis, haemorrhage, and coagulation. The assay was performed in triplicate for both CS and C hydrogel formulations. A positive and a negative control were performed by applying 300 μL of 5 M NaOH and 0.9% NaCl on the CAM, respectively. The irritation score (IS) was used to quantitatively analyse the irritation potential of the tested samples, using Equation (4) [41]:

(4) $$IS=\left(\left(\frac{301-T_{H}}{300}\right)\cdot 5\right)+\left(\left(\frac{301-T_{L}}{300}\right)\cdot 7\right)+\left(\left(\frac{301-T_{C}}{300}\right)\cdot 9\right)$$

where *T_H_*, *T_L_*, and *T_C_* represent the time (in seconds) when the first appearance of haemorrhage, lysis, and coagulation occurs, respectively.

#### 2.6.2. Haemocompatibility

Blood samples were collected from healthy volunteers via venepuncture in sodium citrate anti-coagulant vacutainer tubes (Vacustest Kima, Arzegrande, Italy) under aseptic conditions in Serviços de Saúde of Instituto Superior Técnico, Lisbon, after obtaining informed consent and with the approval of the Ethical Committee of Egas Moniz (Ref. n◦1047/2022). Sterilised disks of both CS and C formulations were placed in falcons containing 5 mL of PBS, under aseptic conditions. A volume of 200 μL of blood was added to the falcons, which were incubated at 37 °C for 1 h. Distilled water and untreated PBS were used as the positive and negative control, respectively. After incubation, the samples were removed from the falcons, and the tubes were centrifuged for 10 min at 3000 rpm. The supernatant’s absorbance (*Abs_sample_*) was measured at 540 nm and related with the absorbance of the positive and negative control (*Abs_C_*_+_ and *Abs_C_*_−_, respectively) through Equation (5) to calculate the haemolysis ratio. The assay was performed in sextuplicate.

(5) $$Hemolysis\;\left(\%\right)=\frac{Abs_{sample}-Abs_{c-}}{Abs_{c+}-Abs_{c-}}\times 100$$

#### 2.6.3. Cytotoxicity

A cytotoxicity assay using NIH/3T3 fibroblasts was performed to evaluate the cells’ response to non-loaded and drug-loaded hydrogels. The assay was performed under sterile conditions (flow chamber from Bio Air Instruments, model AURA 2000 MAC 4 NF), using porous Transwell^®^ inserts (8.0 µm pore polycarbonate membrane Corning^®^ Transwell^®^, Sigma, Saint Louis, MO, USA), and following the ISO-10993-5:2009 guidelines. The cells were cultured in DMEM, and supplemented with 10% bovine calf serum, 1% penicillin-streptomycin solution, and 1% sodium pyruvate. The assays were performed in quadruplicate in 12-well plates. Approximately 1 × 10^5^ cells were seeded in each well in 0.8 mL of DMEM-supplemented medium, corresponding to a cell concentration of 1.25 × 10^5^ cells/mL. The plates were incubated at 37 °C (humidified with 5% CO_2_) for 24 h to promote cell culture and obtain a confluent monolayer. Hydrogel disks (7 mm diameter) were sterilised in PBS or drug solution, using an autoclave (121 °C for 20 min). Thereafter, they were gently blotted with a sterile tissue, and placed in the bottom surface of polycarbonate membrane Transwell^®^ inserts present in the plates. Negative and positive controls were made by supplementing the cells with 1 mL of DMEM, and 1 mL of DMEM with 10% DMSO, respectively. The plates were again incubated for 24 h, and the MTT assay was performed to verify the viability of the cells. After incubation, both the inserts and the medium were carefully removed from the wells, and 300 μL of the MTT solution (10% MTT dissolved in serum-free DMEM) was added. Additional controls without cells were also made and supplemented with the MTT solution. The plates were then incubated for 3 h in the previous incubation conditions. After incubation, 600 μL of the MTT solvent was added to each well, and the plates were agitated on an orbital shaker for 1 h. After dissolution, 200 μL of medium was retrieved from each well, and the absorbance was read at 595 nm in a microplate reader (AMP Platos R 496, AMEDA, Labordiagnostik, Graz, Austria). Cell viability was assessed through relative quantification by normalising it to the negative control.

### 2.7. In Vivo Case Study

The therapeutic efficacy of a drug-loaded hydrogel that was selected after the previous studies was evaluated through one in vivo case study; the case study involved a 13-year-old neutered male dog (Serra da Estrela breed), weighing 30 kg, that was admitted to the veterinary hospital (Hospital Veterinário de S. Bento, Lisbon, Portugal) with multiple dog bite wounds. Physical examination of the dog showed a body condition 3/9, hypotension, prostration, and exudative wounds on the left thoracic limb (two lesions: one caudal and another cranial) and the right pelvic limb (one dorsolateral lesion), with signs of severe pain. The animal had claudication of the left thoracic limb due to a previous identical episode. Biochemical and haemogram analyses were performed and revealed hypoalbuminemia (1.4 g/dL), compatible with protein-losing enteropathy. Initially, the animal required stabilisation with fluid therapy Ringer’s lactate (B. Braun^®^) through a venous catheter in the jugular (Introcan^®^). Analgesic, anti-inflammatory, and antibiotherapy management was performed.

The wounds on the left thoracic limb were cleaned with 1% chlorhexidine (desinclor^®^) until the animal was stable, and cryotherapy was performed two times a day for 20 days. After stabilisation, it was possible to perform trichotomy of the injured areas using an Oster^®^ Golden A5^®^ shearing machine and Oster CryogenX^®^ No. 40 shearing blade. Thereafter, cleaning of the wounds was carried out with isotonic saline Ringer’s lactate (B. Braun^®^) associated with 1% chlorhexidine. Drug-loaded hydrogels dressings (8 × 8 cm^2^) were then applied to the two wounds of the left thoracic limb, gloved, and protected by 100% cotton surgical sterile gauze pads (Bastos Viegas^®^); they were attached to the limb with adhesive (3M™ Durapore™). The dressing was covered with a second layer of elastic bandage (Bastos Viegas^®^) and wrapped in a third layer of self-adhesive bandage (Peha-haht^®^). The procedure was performed in a way to avoid clog of the injured area, ischemia, oedema, or cell death. Dressings were changed daily for the first week and then every 48 h until resolution.

Wound management of the right pelvic limb was performed vi disinfection with 1% chlorhexidine associated with isotonic saline Ringer’s lactate, followed by application of 100% cotton surgical sterile gauze pads (Bastos Viegas^®^) that were fixed to the skin with a non-woven adhesive band (Omnifix^®^E). This management is commonly used in clinical practice in wounds whose closure is expected to occur via second-intention healing, without expected complications.

The study was approved by the Ethical Committee (CEBEA) of Faculdade de Medicina Veterinária da Universidade Lusófona (Ref. n◦27/2019); both the ARRIVE guidelines and EU Directive 2010/63/EU for animal experiments were followed.

### 2.8. Statistical Analysis

Quantitative data are represented as average values and the respective standard deviations. To infer about the statistical significance, statistical tests were performed using the software R Project v. 4.2.1. The Shapiro–Wilk test was used to verify the normality of the data. For data with verified normality, the similarity of variances was evaluated using the Levene’s test. The one-way ANOVA test and Student *t*-test were the parametric tests used to calculate if the sets of data were significantly different. When the equality of variances assumption was not met, the data were analysed by using the Welch’s *t*-test. For non-parametric data, the Kruskal–Wallis test and Wilcoxon signed-rank test were used. The Bonferroni correction was applied for pairwise comparison between groups, as required. The level of significance was set to 0.05.

## 3. Results and Discussion

### 3.1. Hydrogel Characterisation

#### 3.1.1. FTIR Analysis

FTIR spectra of the hydrogels C and CS are compared to those of the pure components (PAAm and casein C and CS) in Figure S1 (Supplementary Information). In the spectra of the hydrogels, bands that are characteristic of both components are visible, but it is not possible to conclude about the eventual interaction between them. In the high-frequency region, the NH_2_ bands at 3338 cm^−1^ and 3159 cm^−1^ characteristic of the primary amide of PAAm and the band 2968 cm^−1^–2873 cm^−1^, deriving from CH_2_ and CH_3_ groups of the casein, clearly identify the presence of these components. The peak corresponding to the stretching of C=O is visible at 1646 cm^−1^, which is a value intermediate between the values of both components. The peak at 1608 cm^−1^ may be attributed to the bending of N-H in primary amide and coincides with the equivalent peak in the PAAm spectrum. The low-frequency region is more difficult to interpret due to the superposition of the peaks in the spectra of both components, although the absence of new peaks seems to indicate no decomposition of the components. Furthermore, we must stress that other components are present in the hydrogels, although in minor quantities, which may contribute to the complexity of the hydrogel spectra.

#### 3.1.2. Swelling Ratio and Equilibrium Water Content

The swelling degree is a critical factor to define the applicability of wound dressings. A dressing with a high SR leads to a good absorption of biological fluids, an efficient nutrient-waste exchange, and favours cell migration [42].

**Figure 2:**
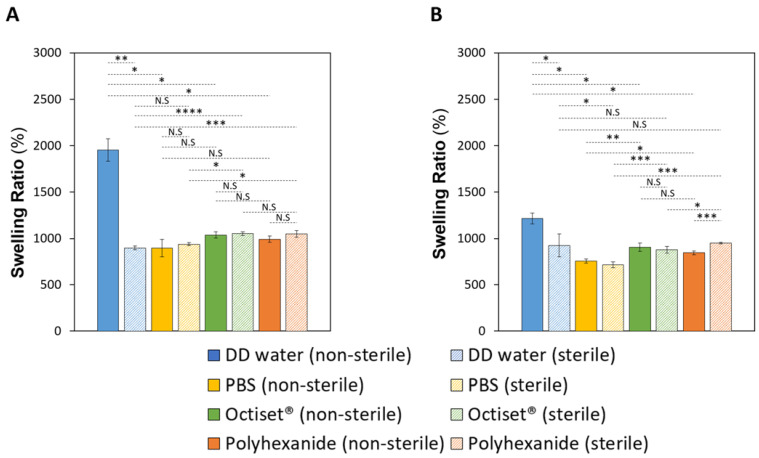
SR of non-sterile and sterile CS (**A**) and C (**B**) hydrogels in DD water, PBS, and drug solutions. The errors are the ± standard deviations (n = 3). Statistical analysis was performed using the Student *t*-test or Wilcoxon signed-rank test, with significance set at * *p* < 0.05, ** *p* < 0.01, *** *p* < 0.005, **** *p* < 0.001. N.S. = not significant.

Figure 2A,B shows the SR of CS and C hydrogel formulations, respectively, before and after sterilisation. Both hydrogels presented very high swelling degrees in DD water, although for formulation C, the value was about 30% lower (*p* < 0.04). A similar behaviour was reported in other studies involving casein hydrogels [43], which may be attributed to the repulsion between the ionic charges in the polymer backbone. In the drug solutions, this repulsion is partially compensated by the attraction of the negative sites in the hydrogels and the positive groups in drug molecules, and the SR decreases although keeping high values. Variation of the ionic strength led to a decrease in the SR value in PBS for both hydrogels (*p* = 0.014 for CS and *p* = 0.019 for C). Concerning sterilisation, the most striking effect was the decrease of SR in DD water (*p* < 0.01 for CS and *p* < 0.02 for C), which was more significant in the case of the CS material; this possibly indicates an increase in the crosslinking degree of the polymer network, which induced the tightening of the hydrogels’ matrix. In contrast, sterilisation did not affect the swelling capacity of both hydrogels in PBS (*p* = 0.608 for CS, *p* = 0.095 for C), Octiset^®^ (*p* = 0.271 for CS, *p* = 0.226 for C), and polyhexanide solution for CS (*p* = 0.061), but a slight increase occurred in this drug for C (*p* < 0.003). A possible explanation is that, in DD water, the effect of the repulsive interactions was attenuated by the increase in pressure and temperature during sterilisation, while in drug solutions, these repulsive interactions were already weak in the non-sterile hydrogels.

The EWC values of the non-sterile hydrogels were within the same range (95.4% ± 0.6 for CS and 92.4% ± 0.3 for C) and decreased slightly to 90.0% ± 0.2 for CS hydrogels, and to 90.2% ± 1.1 for C samples after sterilisation (*p* < 0.001 for CS and *p* < 0.05 for C). The high water content of the dressings allows them to keep a wetted environment in the wound bed, which promotes cellular growth and accelerates the tissue regeneration [44]. Such types of materials are considered suitable for the treatment of a wide a range of wounds, e.g., wounds that leak little or have no exudate as well as those that are painful or necrotic like pressure ulcers, second-degree burns, and infected wounds [45,46].

#### 3.1.3. Degradation Assay

High levels of enzymes like lysozyme and proteases were identified in infected and chronic wounds. The lysozyme concentration has been reported to be 13–24 times higher in infected wound fluid than in uninfected wounds [47,48]. This enzyme, produced by the human immune system, is capable of catalysing the hydrolysis of glycosidic bonds of mucopolysaccharides in bacterial cell walls. Furthermore, it is known that lysozyme can strongly associate with α-casein [49,50,51], and this is the reason for using lysozyme in the degradation assays. Proteases are also quite important in the wound healing process, regulating extracellular matrix degradation and deposition that is critical for wound re-epithelialisation. Proteases break down proteins into peptides and amino acids and can therefore also help destroying casein. Although they have not been used in the present work, their effect involving casein-based dressings will be investigated in future studies, as planned.

**Figure 3:**
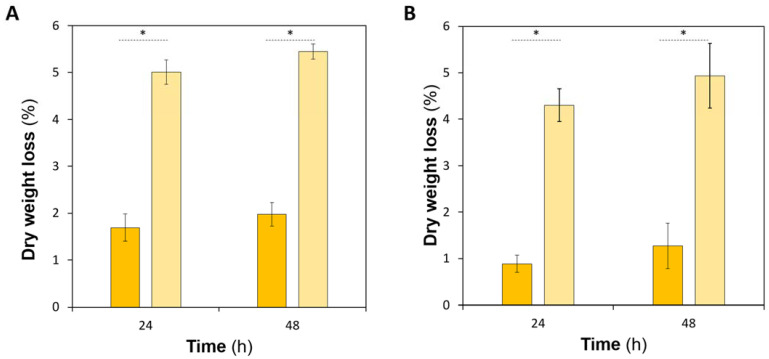
Degradation values for hydrogels of formulation CS (**A**) and C (**B**), in PBS (dark yellow) and in PECF containing lysozyme (light yellow). Error bars correspond to ± standard deviations (n = 4). Statistical analysis was performed using the Student *t*-test or Wilcoxon signed-rank test, with significance set at * *p* < 0.001.

The degradation of the produced hydrogels was determined in PBS and in PECF containing lysozyme at two time points (24 and 48 h, Figure 3).

The hydrogels of both formulations presented very low weight losses in PBS. For formulation CS, the hydrolytic degradation values were 1.6% ± 0.3 and 2.0% ± 0.3 at 24 h and 48 h, respectively. Formulation C materials displayed slightly lower values: 0.9% ± 0.2 at 24 h (*p* < 0.001) and 1.3% ± 0.5 at 48 h (*p* < 0.05).

The degradation values for hydrogels of formulation CS exposed to the PECF + lysozyme solution were 5.0% ± 0.3 and 5.4% ± 0.2 at 24 h and 48 h, respectively. Formulation C materials displayed slightly lower values: 4.3% ± 0.4 at 24 h (*p* < 0.005) and 4.9% ± 0.7 at 48 h (*p* < 0.05). As expected, degradation values of the hydrogels exposed to the simulated exudate solution with lysozyme were greater than those of the hydrolytic degradation (*p* < 0.001 for both hydrogels). A comparison of the two hydrogels shows that the hydrogels of formulation C were slightly more resistant to degradation in both solutions than those of formulation CS, which agrees with the lower swelling capacity of this hydrogel.

#### 3.1.4. SEM

**Figure 4:**
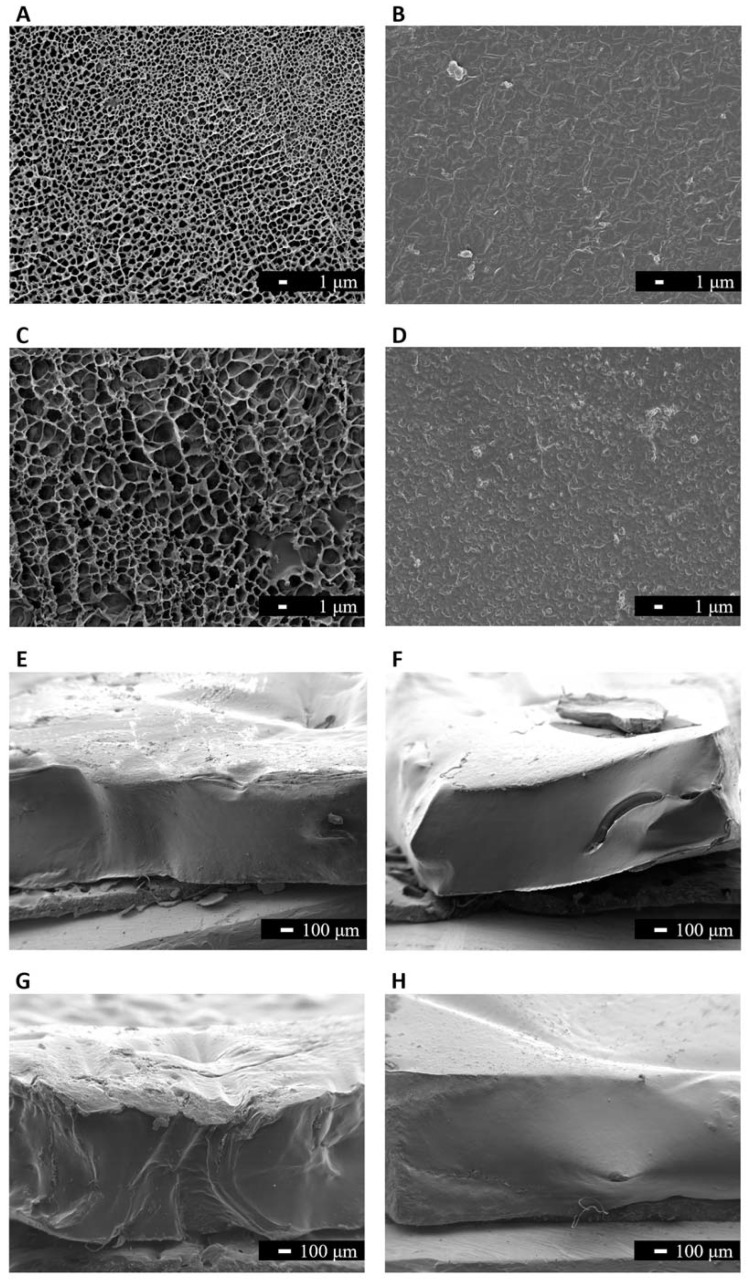
SEM images (3000× magnification, scale bar 1 μm) of the surface of hydrogels with formulation CS (**A**,**B**), and formulation C (**C**,**D**): before sterilisation (**A**,**C**) and after sterilisation (**B**,**D**). Cross-section images (40× magnification, scale bar 100 μm) of the hydrogels of formulation CS (**E**,**F**), and formulation C (**G**,**H**): before sterilisation (**E**,**G**) and after sterilisation (**F**,**H**).

The SEM images of the surfaces of both CS and C materials, before and after sterilisation, are presented in Figure 4A–D. It is possible to identify a lacy structure on the surface of non-sterile samples, which disappeared after sterilisation. Cross-section images of the same materials, before and after sterilisation, are shown in Figure 4E–H. They reveal that the lacy structure results from lumps on the material’s surface and does not correspond to the presence of pores. The harsh conditions of the autoclave process during sterilisation (121 °C, that leads to a water vapour pressure of ≈205 KPa) may induce the collapse of these lacy appearance, inducing a surface smoothing effect.

#### 3.1.5. Mechanical Tests

**Figure 5:**
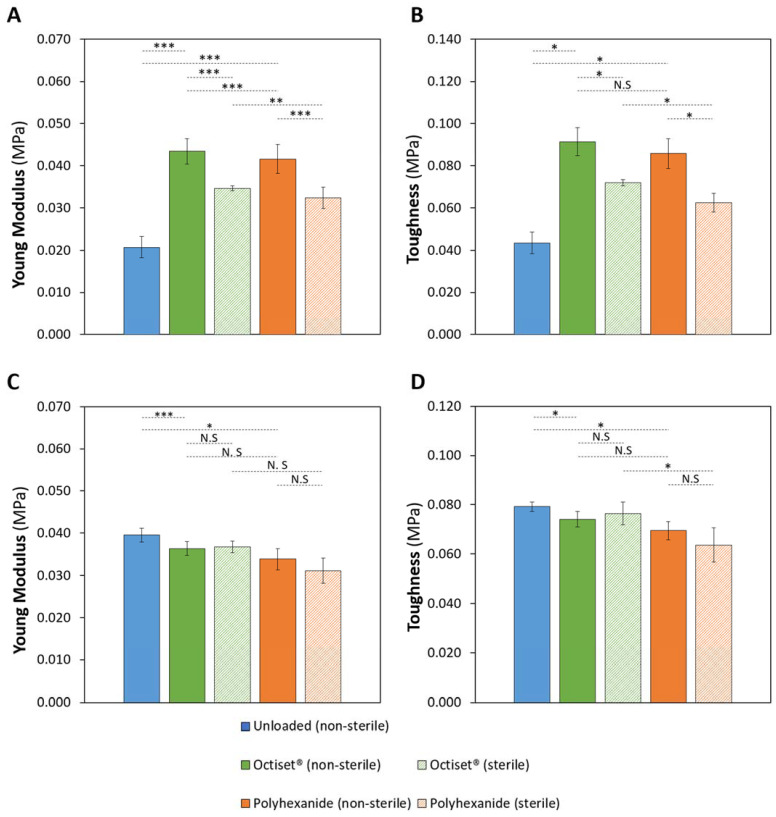
The Young’s modulus (**A**,**C**) and toughness (**B**,**D**) values determined for the strain range 0–20% of non-loaded and drug-loaded sterile and non-sterile CS (**A**,**B**) and C (**C**,**D**) hydrogels. Error bars correspond to ± standard deviations (n = 4). Statistical analysis was performed using the Student *t*-test or Wilcoxon signed-rank test, with significance set at * *p* < 0.05, ** *p* < 0.01, *** *p* < 0.001. N.S. = not significant.

Stress–strain curves of the non-loaded and drug-loaded hydrogels, before and after sterilisation, are shown in Figure S2, respectively, for formulations CS and C. The Young´s modulus and toughness values for CS and C hydrogels were determined in the deformation range 0–20%, where stress and strain are proportionally dependent, and are shown in Figure 5. Concerning non-loaded hydrogels, both values are significantly higher for formulation C, compatible with the lower swelling capacity of this hydrogel and its higher resistance to degradation, which may be attributed to a larger crosslinking degree.

For hydrogels of formulation CS (Figure 5A,B), it is possible to identify a significant increase in the Young´s modulus (*p* < 0.001 for both drugs) and the toughness (*p* < 0.05 for Octiset^®^, *p* < 0.001 for polyhexanide) with drug loading. This implies a less elastic behaviour for the drug-loaded materials, which is in agreement with the lower SR obtained for the CS hydrogels immersed in drug solutions (Figure 2A). Sterilisation led to a slight decrease in both the Young’s modulus (*p* < 0.001 for both drugs) and toughness (*p* < 0.05 for both drugs), which was not reflected in the behaviour of the SR of these hydrogels. In the case of formulation C (Figure 5C,D), the non-loaded and the drug-loaded hydrogels present roughly the same values of the Young´s modulus (*p* = 0.2931 for Octiset^®^, *p* = 0.0508 for polyhexanide) and toughness (*p* = 0.2450 for Octiset^®^, *p* = 0.1620 for polyhexanide), despite the differences observed in their SR (Figure 2B). Sterilisation had practically no effect on the mechanical properties of this formulation.

### 3.2. Drug Loading and Drug Release

The prepared hydrogels were colourless, but after drug loading, they became whitish. The change of colour may be attributed to some drug precipitation inside the polymeric network.

**Figure 6:**
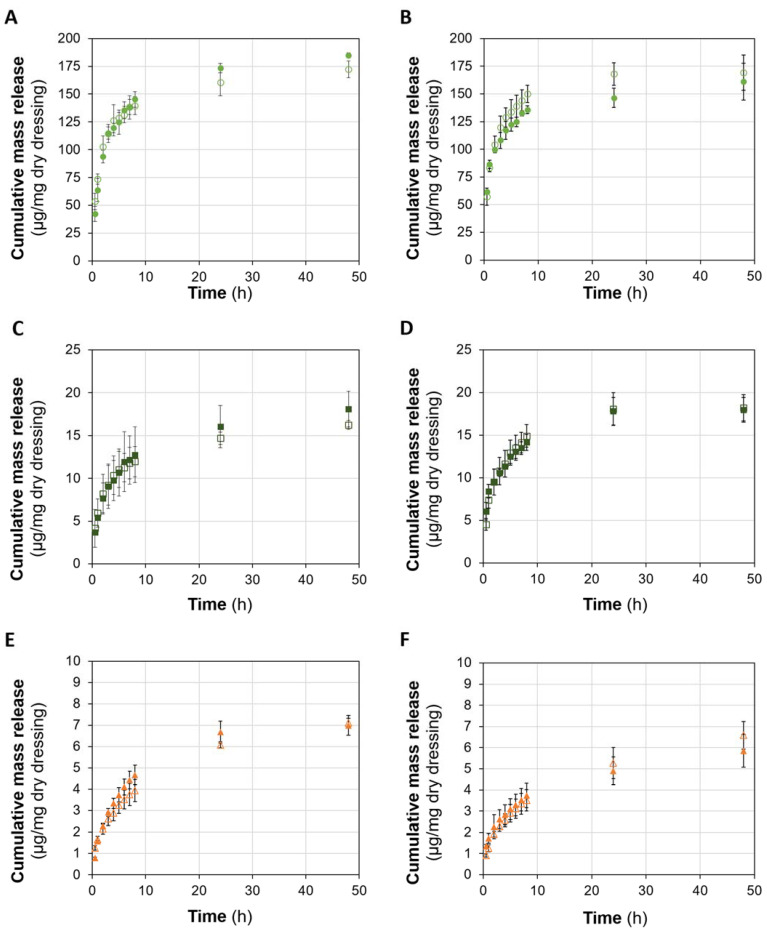
Cumulative drug release profiles from sterile (solid symbols) and non-sterile (open symbols) CS (**A**,**C**,**E**) and C (**B**,**D**,**F**) hydrogels: 2-Phenoxyethanol (**A**,**B**), octenidine dihydrochloride (**C**,**D**), and polyhexanide (**E**,**F**). Error bars correspond to ± standard deviations (n = 3).

The release profiles of the Octiset^®^ components (i.e., 2-phenoxyethanol and octenidine dihydrochloride), and of polyhexanide from the hydrogels with formulation CS and C, both non-sterile and sterile, are presented in Figure 6.

Regarding CS hydrogels, the release of the drugs was in general controlled for at least 48 h. During the first 8 h, the release was fast and then it slowed down between the 24th h and 48th h. Sterilisation did not significantly affect the drug release profiles, which is in agreement with the fact that SR in the drug solutions is almost unaffected by the sterilisation. The results of the methanol extraction assay allowed to conclude that most of the loaded drugs were released. In the case of Octiset^®^-loaded materials, around 75% of 2-phenoxyethanol and around 86% of octenidine dihydrochloride were released in 48 h. This value increased to around 89% for polyhexanide.

For C formulation, the results are similar, except for the Octiset^®^ components, whose release appeared to stop at 48 h. Sterilisation also did not significantly affect the release profiles of the drugs. The percentages of the loaded drugs that were released at 48 h were around 74% for 2 phenoxyethanol, 81% for octenidine dihydrochloride, and 83% for polyhexanide.

### 3.3. Antibacterial Properties

**Figure 7:**
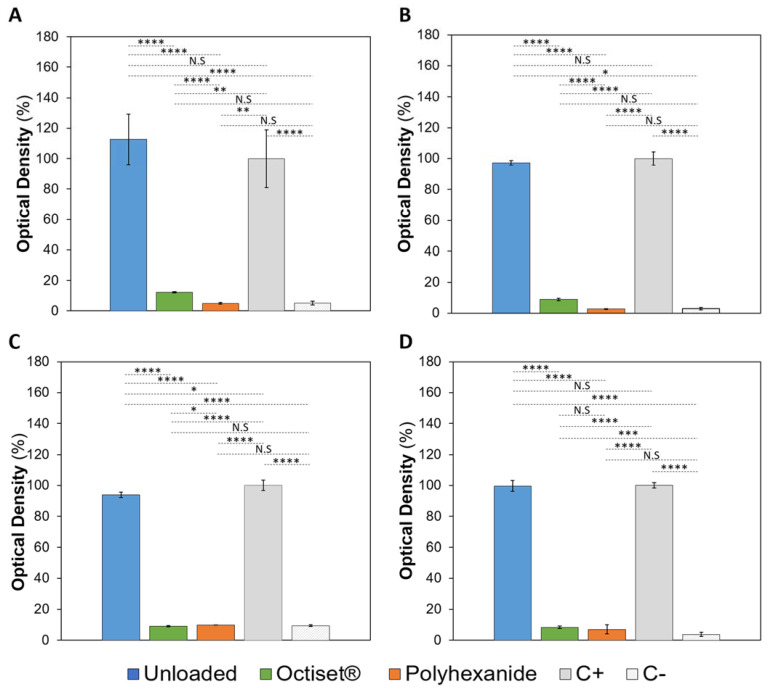
Optical densities of the incubation solutions containing *Staphylococcus aureus* (**A**,**C**) and *Pseudomonas aeruginosa* (**B**,**D**) and non-loaded and drug-loaded CS (**A**,**B**) and C (**C**,**D**) hydrogels. Positive (C+) and negative (C−) controls are also present. The error bars correspond to ± standard deviations (n = 4). Statistical analysis was performed using the Student *t*-test or Wilcoxon signed-rank test, with significance set at * *p* < 0.05, ** *p* < 0.01, *** *p* < 0.005, **** *p* < 0.001. N.S. = not significant.

Figure 7 shows the optical density (%) of the solutions containing *Staphylococcus aureus* and *Pseudomonas aeruginosa* after 24 h incubation of non-loaded and drug-loaded hydrogels with formulations CS and C. The non-loaded samples did not exhibit any antibacterial properties against the tested bacteria. For both formulations, the optical density values of the wells containing those samples were high (being even slightly higher than the value correspondent to the positive control in the case of formulation CS with *Pseudomonas aeruginosa* and C with *Staphylococcus aureus*). Hydrogels of the formulation CS loaded with both drugs demonstrated high antimicrobial activity against *Staphylococcus aureus* and *Pseudomonas aeruginosa*, although polyhexanide-loaded samples appeared to be more effective. For formulation C, the hydrogels loaded with the two drugs also presented high antimicrobial activity, but no significant difference was observed either regarding the strains or the action of the drugs.

### 3.4. Biocompatibility Tests

#### 3.4.1. Irritation Assay (HET-CAM)

**Figure 8:**
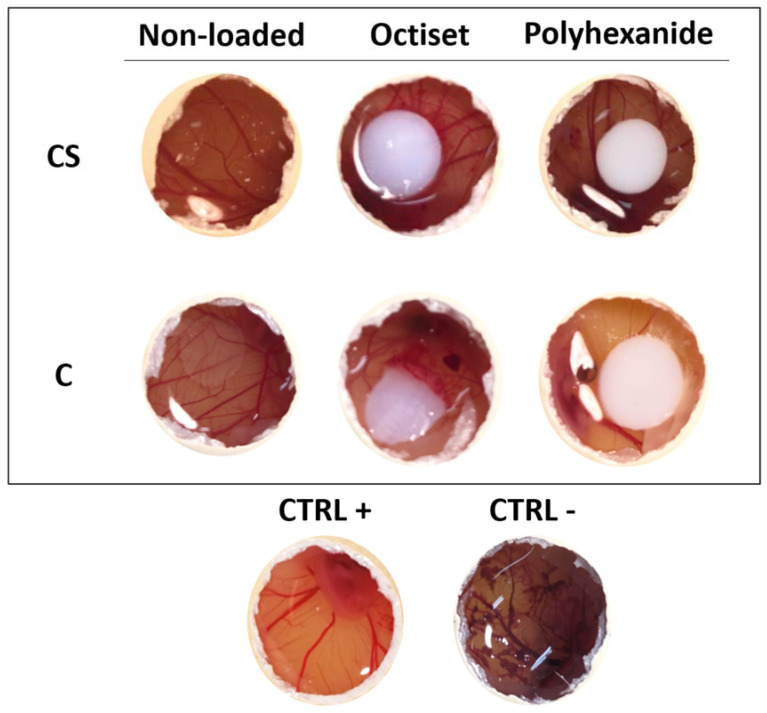
Chorioallantoic membrane images after 5 min contact with CS and C hydrogels, non-loaded and loaded with Octiset^®^ and with polyhexanide. Images of negative control and positive control are included. The non-loaded disks are transparent, while the drug-loaded ones are whitish.

The HET-CAM assay provides a highly used and well-established prediction model for eye irritation, but it can also be performed to evaluate the irritability wound care systems. The results are shown in Figure 8.

The results were very similar for both formulations. Non-loaded hydrogels did not show any signs of membrane of lysis, haemorrhage, or coagulation (IS = 0), as observed in the negative control. The same was true for polyhexanide-loaded ones. The membranes in contact with Octiset^®^-loaded hydrogels of both formulations presented with signs of slight irritation (IS = 1).

#### 3.4.2. Haemocompatibility

The haemocompatibility of the dressing materials is important to avoid an undesired immune response when the materials enter in contact with blood. A haemolysis index lower than 5% corresponds to a highly haemocompatible material; within 10% haemolysis, the material is considered haemocompatible, and for a haemolysis ratio higher than 20%, the material is non-haemocompatible [52,53]. The haemolysis ratios (Figure S3) of the hydrogels were 0.4 ± 0.1 for formulation CS and 0.3 ± 0.1 for formulation C, meaning that both formulations are highly haemocompatible.

#### 3.4.3. Cytotoxicity

**Figure 9:**
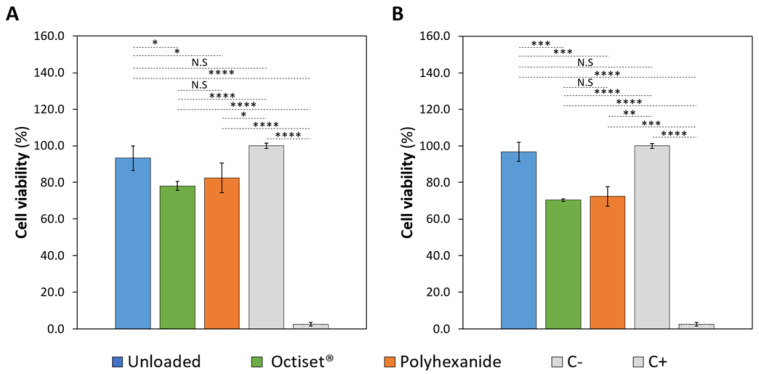
Viability of fibroblasts after growth in culture medium containing non-loaded and drug-loaded hydrogels with formulation CS (**A**) and C (**B**). Error bars correspond to ± standard deviations (n = 4). Statistical analysis was performed using the Student *t*-test or Wilcoxon signed-rank test, with significance set at * *p* < 0.05, ** *p* < 0.01, *** *p* < 0.005, **** *p* < 0.001. N.S. = not significant.

In wound dressings, cytotoxic effects would impair the viability, proliferation, and migration of cells involved in wound healing, thereby lowering the healing rate [54]. The MTT assay allows the measurement of cellular metabolic activity as an indicator of cell viability. The obtained cell viability results are presented in Figure 9.

It is important to point out that, as stated in ISO 10993-5:2009 [55], a material can be considered non-cytotoxic if the cell viability is above 70%. The results obtained for both non-loaded hydrogels show that they are not cytotoxic: around 93.2% of cell viability for CS and 96.7% for the C formulation. Regarding the Octiset^®^-loaded samples, cell viability values were 78% for CS and 70.5% for C. These results are compatible with the clinical and experimental evidence showing that octenidine-containing products are effective anti-bacterial agents, without compromising wound healing or causing significant cytotoxicity [56]. Polyhexanide-loaded hydrogels also displayed non-cytotoxicity with 82.4% cell viability for the CS formulation and 72.5% for the C formulation. Again, this is in agreement with literature reports about the safety, efficacy, and tolerability of polyhexanide [57].

### 3.5. In Vivo Case Study

C hydrogels loaded with Octiset^®^ were used in the in vivo study. Although the formulations CS and C showed similar results, the facts that the preparation of the latter is much faster (6 h vs. 22 h) and its degradation is slightly lower justified the selection of C hydrogels for this study. The antimicrobial tests (Section 3.3) showed that the performance of C hydrogels loaded either with Octiset^®^ or with polyhexanide was equivalent. In a comparative study of different antiseptics, Koburger et al. [26] concluded that polyhexanide and octenidine solutions had similar efficacy when a prolonged contact time with the tissues was feasible, which is the case of wound dressing treatments. Octiset^®^ was chosen because it is a new generation antiseptic that has broad-spectrum efficacy and no known microbial resistance, similar to polyhexanide. It is safe and well tolerated, has no side effects, and is not absorbed systemically, in contrast to polyhexanide that has a high affinity for tissue structures that impairs its long-term use [58].

**Figure 10:**
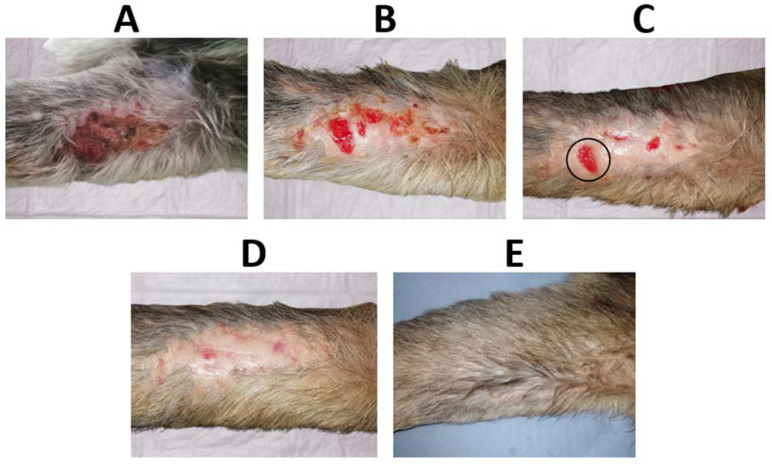
(**A**) Initial cranial wound on the left thoracic limb (untreated, 1st day), (**B**) wound on day 4, (**C**) wound on day 7, (**D**) wound on day 13, and (**E**) wound after 56 days of treatment with the antiseptic-loaded casein hydrogel. The wound limited by the black circle in (**C**) will be compared with a similar wound in Figure 12.

**Figure 11:**
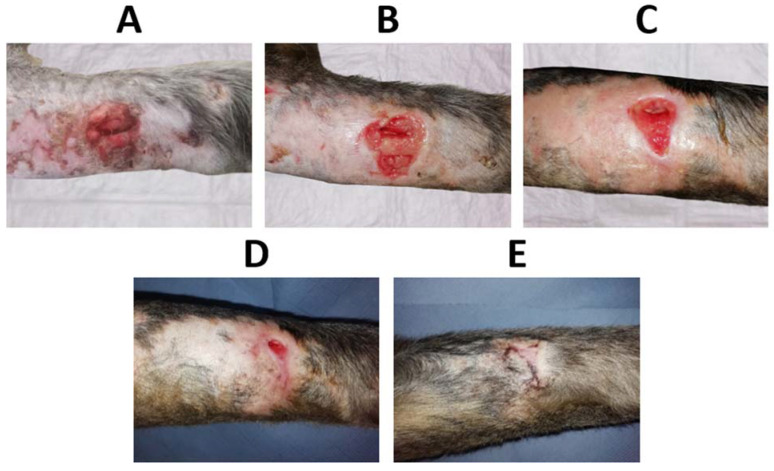
(**A**) Initial caudal wound on the left thoracic limb (untreated, 1st day), (**B**) wound on day 4, (**C**) wound on day 13, (**D**) wound on day 25, and (**E**) wound after 56 days of treatment with the antiseptic-loaded casein hydrogel.

The in vivo performance of C hydrogels dressings loaded with Octiset^®^ was evaluated through the evolution of the healing of the wounds on the left thoracic limb. Figure 10 shows the evolution of the cranial lesion (≈65 mm × 30 mm) in the region of the radius and ulna, which was quite remarkable. The dressings were changed daily for the first week and then every 48 h until resolution. It took only 11 days for epithelialisation to occur along the entire length of the wound. After 56 days, hair follicle growth was observed, and tissue remodelling led to an increase in the tension strength exhibited by the scar, which was not weakened. The evolution of the caudal lesion (≈45 mm × 20 mm), also in the region of the radius and ulna in the same limb, is displayed in Figure 11. Epithelialisation took 34 days to complete due to its high depth (≈10 mm) and tissue trauma. After 56 days, follicular growth was observed in part of the scar, and the initial area with greater depth of tissue damage did not show any follicular growth. Hyperpigmented areas due to melanin deposition were also observed after the complete closure of the lesion. Throughout the process, the dressing properties promoted autolytic debridement, allowing the removal of exudate, necrotic, and devitalised tissue present in the lesions, and no complications were observed during the healing course.

**Figure 12:**
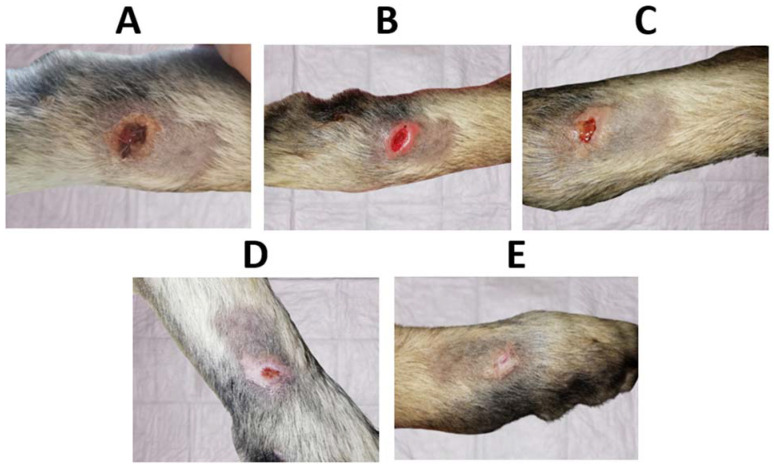
(**A**) Initial dorsolateral wound on the right pelvic limb, (**B**) wound on day 5, (**C**) wound on day 9, (**D**) wound on day 11, and (**E**) wound after 15 days of treatment without antiseptic-loaded casein hydrogels.

In order to compare the time to closure between a wound where C hydrogel dressings loaded with Octiset^®^ were applied and another wound where only daily disinfection was performed and covered with a cotton pad, two wounds were considered: the cranial lesion of the left thoracic limb (Figure 10) and the dorsolateral lesion of the right pelvic limb (Figure 12). On day 7 of the treatment, the dimensions of the cranial wound limited by the black circle in Figure 10C (≈14 mm × 11 mm) were similar to those of caudal wound on day 5 of treatment (Figure 12B) (≈13 mm × 11 mm). Both lesions were in the same healing phase. The former one healed on day 13 of treatment while the latter healed on day 15. This analysis shows that the evolution of the cranial wound in the period of days 7–13 is equivalent to that of the caudal wound in the period of days 5–15, leading to the conclusion that the use of the dressings accelerated the healing process by 4 days.

## 4. Conclusions

The main goal of the present work was to investigate the possibility of developing novel casein hydrogel dressings to treat chronic wounds. Additionally, these dressings were loaded with antiseptics, aiming to ensure a more rapid and efficient wound care treatment. Two casein hydrogel formulations were tested: casein sodium salt (CS) and acid casein (C). The hydrogels presented with high swelling capacity, low degradation in simulated exudate solution, and adequate mechanical properties to be used as wound dressings. Although the properties of both hydrogels were similar, some differences were found. The hydrogel of formulation C was characterised by a lower SR, a lower elasticity, and a higher resistance to degradation. Autoclave sterilisation did not affect the properties of both hydrogels. When loaded with Octiset^®^ or polyhexanide, the hydrogels were able to release the drugs in a sustained manner for, at least, 48 h. Both antiseptic-loaded materials presented good antimicrobial properties and were demonstrated to be non-irritant, highly haemocompatible, and non-cytotoxic. A case study involving a dog with multiple wounds was conducted. Three-layer dressings based on casein hydrogels (formulation C) loaded with Octiset^®^ led to an efficient healing process. Altogether, the obtained results indicate that the developed casein hydrogels appear to be promising wound dressing materials.

## Data Availability

Data available upon request.

## Supplementary Materials

The following supporting information can be downloaded at: www.mdpi.com/article/10.3390/pharmaceutics15020334/s1, Figure S1: FTIR-ATR spectra of the non-loaded and non-sterile CS (A) and C (B) hydrogels, in the region of 4000–900 cm^−1^; Figure S2: Stress-strain curves of non-loaded and Octiset^®^ (A,C) and polyhexanide (B,D) loaded CS (A,B) and C (C,D) formulation samples, before and after sterilisation. Error bars represent the maximum standard deviations (n = 4); Figure S3: Hemolysis ratios for formulation CS and C. Error bars correspond to ± standard deviations (n = 4). Statistical analysis was performed by Student-t, which significance set at * *p* < 0.001.
