# Supplementary material for: Antiseptic-Loaded Casein Hydrogels for Wound Dressings
Source: Pharmaceutics. 2023 Jan 19;15(2):334. doi: 10.3390/pharmaceutics15020334 (PMC9967843; doi:10.3390/pharmaceutics15020334)
Supplement: Supplementary file 1 [file pharmaceutics-15-00334-s001.zip › pharmaceutics-2124928-supplementary.pdf]

Supporting information

Antiseptic-Loaded Casein Hydrogels for Wound Dressings

Leonor Vasconcelos Garcia [1], Diana Silva [1,*], Maria Madalena Costa [2,3], Henrique Armés [2,3], Madalena Salema-Oom [4], Benilde Saramago [1,*] and Ana Paula Serro [1,4]

[1] Centro de Química Estrutural, Institute of Molecular Sciences, Departamento de Engenharia Química, Instituto Superior Técnico, Universidade de Lisboa, Av. Rovisco Pais, 1049-001 Lisboa, Portugal
[2] Hospital Veterinário de S. Bento, Rua de S. Bento, 358-A, 1200-822 Lisboa, Portugal
[3] Faculdade de Medicina Veterinária, Universidade Lusófona, Campo Grande, 376, 1749-024 Lisboa, Portugal
[4] Centro de Investigação Interdisciplinar Egas Moniz (CiiEM), Instituto Universitário Egas Moniz, 2829-511 Caparica, Portugal
* Correspondence: dianacristinasilva@tecnico.ulisboa.pt (D.S.); b.saramago@tecnico.ulisboa.pt (B.S.)

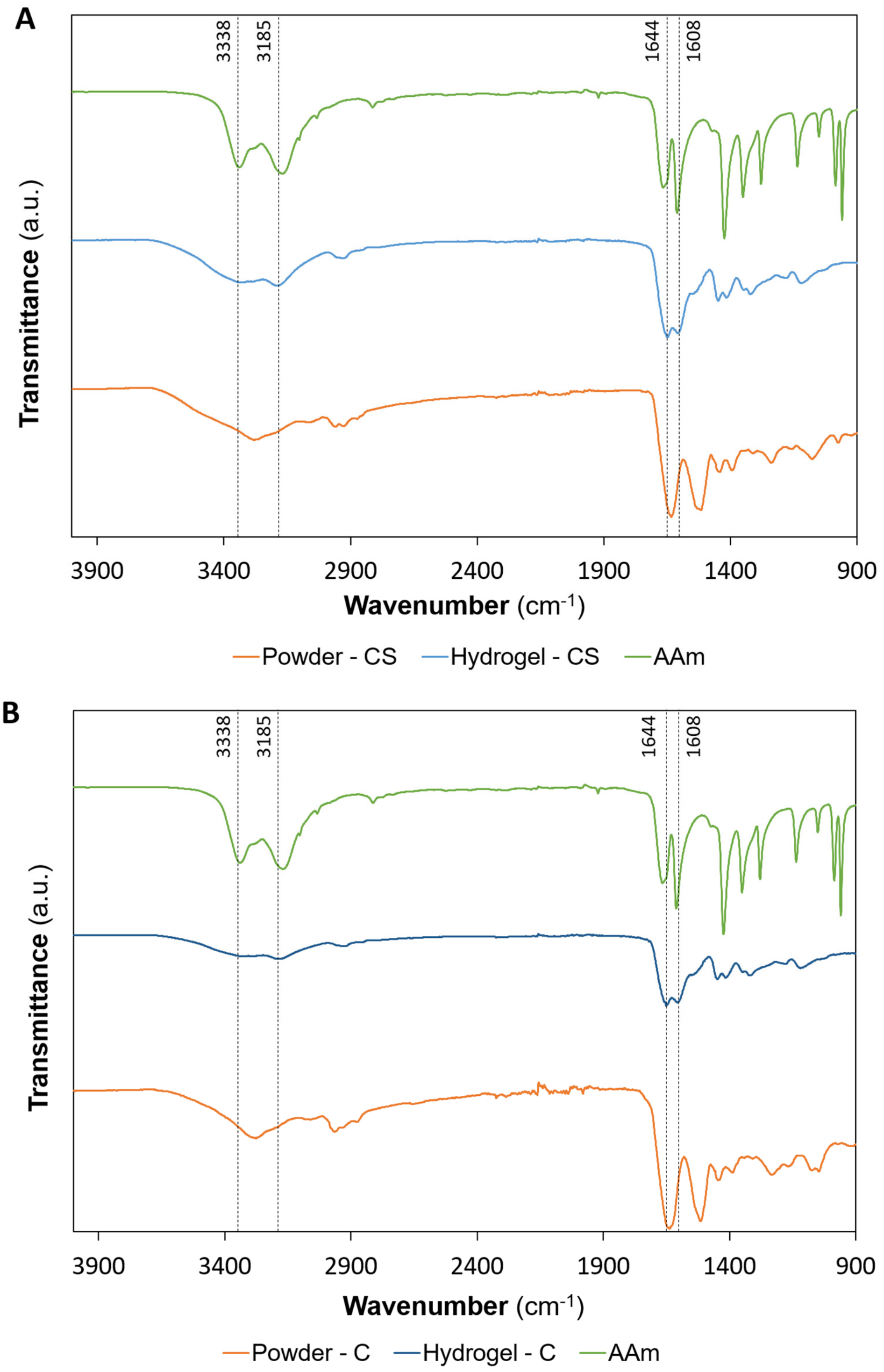

Figure S1. FTIR-ATR spectra of the non-loaded and non-sterile CS (A) and C (B) hydrogels, in the region of 4000–900 cm^{-1}.

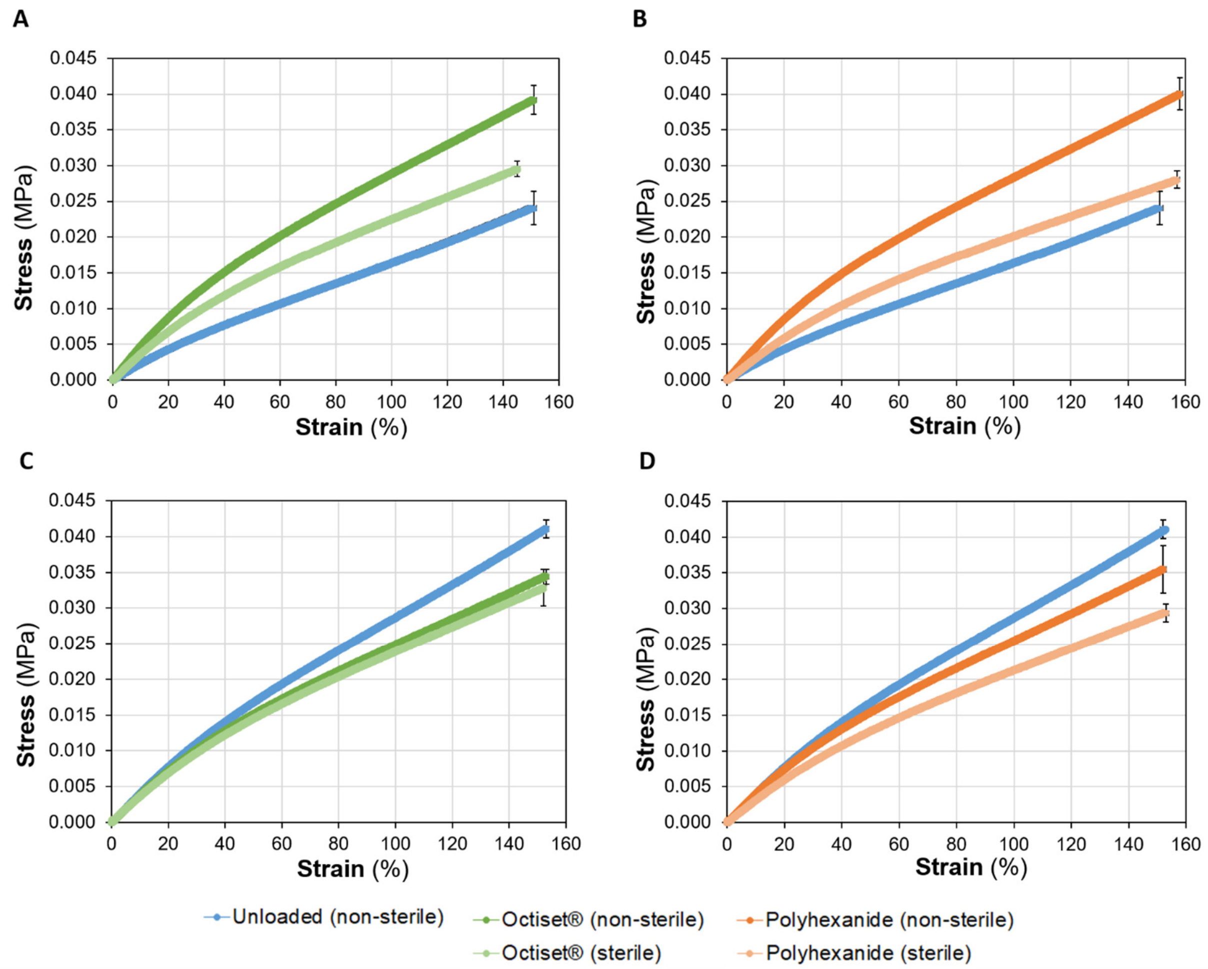

Figure S2. Stress-strain curves of non-loaded and Octiset® (A, C) and polyhexanide (B, D) loaded CS (A, B) and C (C, D) formulation samples, before and after sterilisation. Error bars represent the maximum standard deviations (n=4).

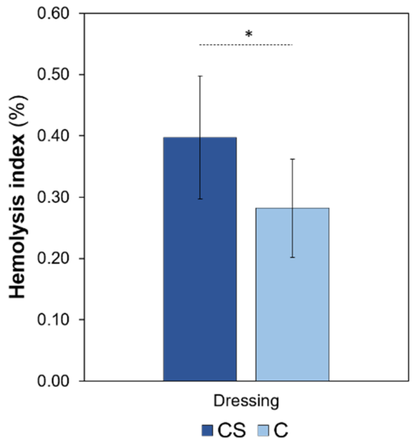

Figure S3. Hemolysis ratios for formulation CS and C. Error bars correspond to ± standard deviations (n=4). Statistical analysis was performed by Student-t, which significance set at *p < 0.001.
